# Proposition of zinc supplementation during levodopa–carbidopa intestinal gel treatment

**DOI:** 10.1002/brb3.1143

**Published:** 2018-10-25

**Authors:** Hirofumi Matsuyama, Keita Matsuura, Hidehiro Ishikawa, Yoshinori Hirata, Natsuko Kato, Atsushi Niwa, Yugo Narita, Hidekazu Tomimoto

**Affiliations:** ^1^ Department of Neurology Mie University Graduate School of Medicine Tsu Japan

**Keywords:** levodopa–carbidopa intestinal gel, Parkinson's disease, zinc deficiency

## Abstract

**Objectives:**

Levodopa–carbidopa intestinal gel (LCIG) infusion is a useful therapy for the wearing‐off phenomenon of advanced Parkinson's disease (PD) patients. Recently, we found three PD patients that may have had a zinc deficiency after the LCIG infusion, possibly due to the zinc‐chelating action of levodopa. This study aims to evaluate changes in serum zinc levels in three patients that received LCIG treatment and to determine possible remedies for zinc deficiency during treatment.

**Materials and Methods:**

We performed a prospective blood analysis of serum zinc levels before, when possible, and after LCIG treatment in our three PD patients.

**Results:**

The serum zinc levels of the first patient before treatment and 4 months after beginning LCIG treatment were 69 and 58 μg/dl, respectively. For the second patient, serum zinc levels before treatment and two months after starting LCIG treatment were 87 and 46 μg/dl, respectively. The baseline serum zinc level for the third patient was not examined, but was 48 μg/dl 5 months after starting the LCIG infusion.

**Conclusions:**

Levodopa–carbidopa intestinal gel infusion might have caused a zinc deficiency through levodopa zinc chelation. Zinc deficiency with LCIG infusion has not yet been reported, though preventing zinc deficiency may be an important factor in future LCIG treatment strategies.

## 1 INTRODUCTION

The elderly population in Japan is growing, and applicability of device‐aided treatment is increasing respectively. Levodopa–carbidopa intestinal gel (LCIG) infusion treatment involves a device for treating the wearing‐off phenomenon in advanced‐stage Parkinson's disease (PD) patients. This treatment was approved in Japan in July 2016 for advanced‐stage PD patients having difficulty with peroral medications. LCIG is widely prescribed to control motor fluctuations in PD patients and administered through a percutaneous gastrojejunostomy (PEG‐J; Lopiano et al., 2016). It is believed that introducing LCIG significantly improves specific nonmotor symptoms in advanced PD patients, enhances quality of life, and facilitates daily living activities (Krüger et al., 2017). Furthermore, a 2‐year observational study of the clinical safety and effectiveness of LCIG showed that the treatment significantly shortened off‐time, decreased dyskinesia, and improved nonmotor symptom in advanced PD patients (Antonini et al., 2017).

Levodopa–carbidopa intestinal gel treatment utilizes levodopa (L‐dopa), a reported zinc chelator. Long‐term intake of drugs which have a potential to chelate zinc may cause a zinc deficiency. After L‐dopa binds to internal zinc, the compound is excreted in urine; over time, this process can lead to a zinc deficiency (Tomita & Yoshikawa, 2002). Recently, a taste disorder due to zinc deficiency in the elderly people has been observed; zinc deficiencies may also cause bedsores and skin diseases which in turn could impact wound healing after the PEG‐J operation (Tasaki, Hanada, & Hashimoto, 1993). Since zinc deficiency is likely with LCIG infusions, but such a correlation has not yet been reported.

## 2 METHODS

We investigated serum zinc levels by a prospective blood analysis before, when possible, and after LCIG treatment in three PD patients admitted to the Mie University Hospital from August 2017 to February 2018. For PD, we used the criteria for clinically probable PD of the Movement Disorder Society Clinical Diagnostic Criteria for PD (Postuma et al., 2015). Informed consent was obtained from all patients in accordance with the Declaration of Helsinki.

## 3 RESULTS

**Figure 1:**
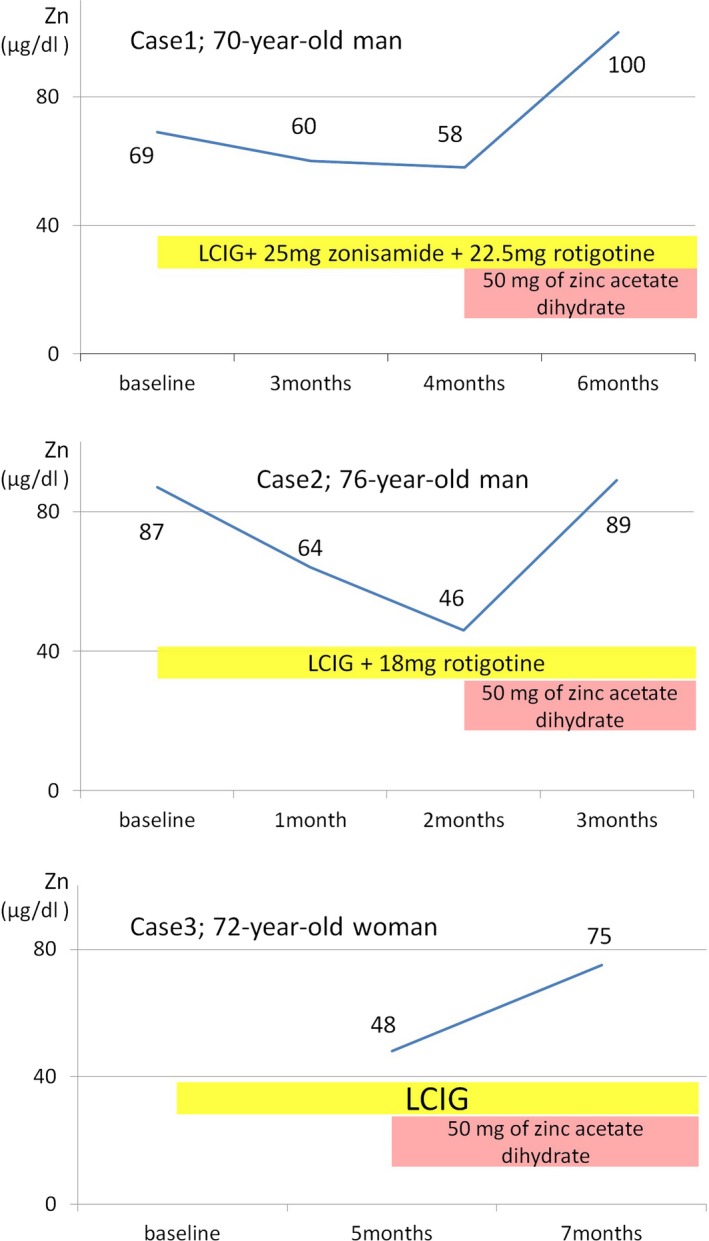
Clinical course of our three cases showing serum zinc levels, treatment of Parkinson's disease (PD), and zinc supplemental therapy

The first patient was a 70‐year‐old male who developed small‐step gait at age 48 (Figure 1). At age 66, he developed wearing‐off and on‐off symptoms. In addition, he showed dyskinesia, dementia, and had an anxiety disorder characterized by off‐time panic attacks. Before starting continuous infusion of LCIG, he was estimated to be stage IV on the Hoehn–Yahr scale (HY‐S). His treatment consisted of 650 mg levodopa–decarboxylase inhibitor (L‐dopa/DCI), 500 mg entacapone, 25 mg zonisamide, and a rotigotine dosage of 10 mg/24 hr. Since he responded favorably to LCIG administered through a temporary nasoduodenal/nasojejunal tube, we introduced a permanent PEG‐J in September 2017. The International Parkinson and Movement Disorder Society Unified Parkinson's Disease Rating Scale (MDS‐UPDRS) part III scores were 33 at baseline and 20 three months after the start of LCIG treatment. Zinc levels at baseline, 3 months, and 4 months before the start of LCIG treatment were 69, 60, and 58 μg/dl, respectively. We started a 50 mg supplement of zinc acetate dihydrate, improving the zinc level improved to 100 μg/dl and reducing fatigue 6 months after the start of LCIG.

The second patient was a 76‐year‐old male who developed a left limb resting tremor at age 60 (Figure 1). Ten years later, he developed wearing‐off symptoms and dyskinesia without dementia. His motor disturbance in off‐time gradually increased. At age 75, his motor function deteriorated further after a urinary tract infection and he requested device treatment for PD. He was classified as HY‐S stage III and was treated with 600 mg levodopa/carbidopa hydrate (L‐dopa/CH), 500 mg entacapone, and a rotigotine dosage of 16 mg/24 hr. The MDS‐UPDRS part III baseline scores for on‐time and off‐time were 29 and 71, respectively. We introduced PEG‐J in February of 2018; motor symptom fluctuation was nearly eliminated after LCIG treatment, so we reduced the rotigotine dosage. His serum zinc levels at baseline and 2 months after introduction of LCIG treatment were 87 and 46 μg/dl, respectively. Once we started 50 mg of zinc acetate dihydrate, his zinc level improved to 89 μg/dl after 3 months of LCIG treatment.

The third patient was a 72‐year‐old female who developed gait disturbance at age 64 and a left limb resting tremor at age 66 (Figure 1). At age 69, she developed wearing‐off symptoms including hallucinations gait freezing. She was classified as HY‐S stages III‐IV and was treated with 500 mg L‐dopa/CH, 500 mg entacapone, and a rotigotine dosage of 18 mg/24 hr. Subsequently, we discontinued rotigotine due to hallucinations. The MDS‐UPDRS part III scores for on‐time and off‐time at baseline were 36 and 63, respectively. When we introduced LCIG through PEG‐J on August 31, 2017, her fluctuation of motor symptom improved. Unfortunately, we did not measure the zinc level of baseline, but confirmed a significant zinc deficiency (48 μg/dl) 5 months after introducing LCIG. We started 50 mg of zinc acetate dihydrate, and her zinc level improved to 75 μg/dl after seven months of LCIG treatment.

## 4 DISCUSSION

All three patients who received LCIG treatment had decreased serum zinc values and required continuous zinc supplementation. Unfortunately, there were no data for baseline zinc levels in the third case, but all patients showed abnormally low zinc levels during LCIG treatment and recovered after supplementation. It is therefore postulated that LCIG treatment causes a zinc deficiency. Clinical symptoms of a zinc deficiency include a taste disorder, anorexia, glossodynia, bedsore, and skin disease, but none of the three patients exhibited such symptoms. However, the first patient reported reduced fatigue after receiving zinc supplementation. In previous studies, zinc deficiency has been observed in the cerebrospinal fluid, but not in blood samples of PD patients with L‐dopa treatment (Qureshi, Qureshi, Memon, & Parvez, 2006); therefore, we need more information on the significance of zinc deficiency in the blood samples which may determine the need for zinc supplementation during LCIG treatment.

In our study, chelating action by L‐dopa used in LCIG treatment is considered to cause zinc deficiency in the cases presented. Comorbidities or underlying conditions that could also cause zinc deficiency include liver disease (Sengupta et al., 2015), diabetes (Walter et al., 1991), chronic inflammatory bowel disease (Naber, van den Hamer, Baadenhuysen, & Jansen, 1998), and renal diseases such as the nephrotic syndrome and malabsorption after the small intestine excision (Makhlough et al., 2015). The first patient has diabetes mellitus but is unlikely the sole cause for zinc deficiency. Furthermore, it has been reported that some drugs possess zinc‐chelating capability and may be the reason for drug‐related taste diabetes mellitus disturbances (Tomita & Yoshikawa, 2002). It has been reported that L‐dopa may induce zinc deficiency (Tomita & Yoshikawa, 2002), which chelates zinc (Veldkamp, Tubergen, Swartz, DeVries, & Tatko, 2017). Prior to this study, no reports showed serum zinc levels decreasing progressively with LCIG treatment. As a cause of the change in the profile of zinc chelation, it is plausible that intestinal mucosa ability to absorb zinc may be disturbed by continuous administration of L‐dopa. Because we only changed oral L‐dopa to LCIG in each case, we consider that there is little influence of other confounders which may cause zinc levels. However, we cannot deny the possibility that a drug except for L‐dopa influenced zinc level.

Our study has some limitations. First, the sample size was too small to get statistical power and also detailed information on the effects of zinc deficiency. We have not experienced cases with peripheral neuropathy, but in previous studies, it has been reported to occur as a side effect of LCIG treatment, and homocysteine level and high dose L‐dopa were correlated to chronic peripheral neuropathy (Merola et al., 2014, 2016). Obviously, it is important to accumulate more number of cases in the future. The second point includes a lack of comparison with the treatment group by oral L‐dopa treatment. It is important to know the expectancy of zinc deficiency in LCIG treatment or alternatively, in L‐dopa treatment, and should be delineated in the future studies.

In this study, we showed that LCIG treatment may lead to zinc deficiency in PD patients and suggest including zinc supplements with LCIG treatments. In past studies, vitamin B deficiencies that may cause peripheral neuropathy have been shown after long‐term LCIG infusions (Rispoli et al., 2017). Therefore, vitamin B supplementation, in addition to zinc supplementation, may be important for patients receiving LCIG treatment.

## CONFLICT OF INTEREST

Authors declare no conflict of interest.
